# Prognostic models for surgical-site infection in gastrointestinal surgery: systematic review

**DOI:** 10.1093/bjs/znad187

**Published:** 2023-07-12

**Authors:** Kenneth A McLean, Tanvi Goel, Samuel Lawday, Aya Riad, Joana Simoes, Stephen R Knight, Dhruva Ghosh, James C Glasbey, Aneel Bhangu, Ewen M Harrison, NIHR Global Health Research Unit on Global Surgery

**Affiliations:** Department of Clinical Surgery, Royal Infirmary of Edinburgh, Edinburgh, UK; India Hub, NIHR Global Health Research Unit on Global Surgery, Ludhiana, India; Bristol Centre for Surgical Research, University of Bristol, Bristol, UK; Department of Clinical Surgery, Royal Infirmary of Edinburgh, Edinburgh, UK; Institute of Translational Medicine, University of Birmingham, Birmingham, UK; Department of Clinical Surgery, Royal Infirmary of Edinburgh, Edinburgh, UK; India Hub, NIHR Global Health Research Unit on Global Surgery, Ludhiana, India; Institute of Translational Medicine, University of Birmingham, Birmingham, UK; Institute of Translational Medicine, University of Birmingham, Birmingham, UK; Department of Clinical Surgery, Royal Infirmary of Edinburgh, Edinburgh, UK; Centre for Medical Informatics, Usher Institute, University of Edinburgh, Edinburgh, UK

## Abstract

**Background:**

Identification of patients at high risk of surgical-site infection may allow clinicians to target interventions and monitoring to minimize associated morbidity. The aim of this systematic review was to identify and evaluate prognostic tools for the prediction of surgical-site infection in gastrointestinal surgery.

**Methods:**

This systematic review sought to identify original studies describing the development and validation of prognostic models for 30-day SSI after gastrointestinal surgery (PROSPERO: CRD42022311019). MEDLINE, Embase, Global Health, and IEEE Xplore were searched from 1 January 2000 to 24 February 2022. Studies were excluded if prognostic models included postoperative parameters or were procedure specific. A narrative synthesis was performed, with sample-size sufficiency, discriminative ability (area under the receiver operating characteristic curve), and prognostic accuracy compared.

**Results:**

Of 2249 records reviewed, 23 eligible prognostic models were identified. A total of 13 (57 per cent) reported no internal validation and only 4 (17 per cent) had undergone external validation. Most identified operative contamination (57 per cent, 13 of 23) and duration (52 per cent, 12 of 23) as important predictors; however, there remained substantial heterogeneity in other predictors identified (range 2–28). All models demonstrated a high risk of bias due to the analytic approach, with overall low applicability to an undifferentiated gastrointestinal surgical population. Model discrimination was reported in most studies (83 per cent, 19 of 23); however, calibration (22 per cent, 5 of 23) and prognostic accuracy (17 per cent, 4 of 23) were infrequently assessed. Of externally validated models (of which there were four), none displayed ‘good’ discrimination (area under the receiver operating characteristic curve greater than or equal to 0.7).

**Conclusion:**

The risk of surgical-site infection after gastrointestinal surgery is insufficiently described by existing risk-prediction tools, which are not suitable for routine use. Novel risk-stratification tools are required to target perioperative interventions and mitigate modifiable risk factors.

## Teaser

The aim of this systematic review was to identify original studies describing the development and validation of prognostic models for 30-day surgical-site infection after gastrointestinal surgery. The risk of surgical-site infection after gastrointestinal surgery is insufficiently described by existing risk-prediction tools, which are not suitable for routine use. Novel risk-stratification tools are required to target perioperative interventions and mitigate modifiable risk factors.

## Introduction

Surgical-site infection (SSI) represents the most common complication after gastrointestinal surgery, affecting as many as one in nine patients in high-income countries and one in three patients in low- and middle-income countries^1^. Reducing the incidence and severity of SSI remains a high-priority issue for patients, surgical teams, and healthcare systems^2,3^, due to the substantial contribution of SSI towards postoperative morbidity and mortality^1,4^, quality of life^5^, and healthcare costs^6^.

The capability to accurately predict patients who are at high risk of SSI has several potential advantages. At an individual level, this would allow individualized preoperative assessment of the risk of SSI and prioritization of evidence-based interventions could lead to iatrogenic harm (for example antibiotic prophylaxis) or are resource intensive (for example increased postoperative monitoring) towards patients at highest risk^7^. However, there are also wider benefits, including improving the efficiency of clinical trials on SSI by facilitating the selection of patients most likely to benefit from the trial intervention^8^ and allowing risk adjustment to facilitate the fair comparison of SSI rates across different sites and populations^9^.

However, while clinical risk-prediction tools have increasingly been developed across all areas of medicine, frequently these fail to align with methodological recommendations^10^. They often lack validation outside the original cohorts, meaning their clinical utility remains uncertain^11^. Efforts to develop predictive tools for SSI have been ongoing for decades^12,13^, yet none has been widely adopted to predict individual risk for patients undergoing gastrointestinal surgery^7^. Furthermore, to the best of the authors’ knowledge, no previous systematic reviews have been conducted to determine what models have been developed and if these are suitable for wider adoption. Therefore, the aim of this systematic review was to identify and assess the quality of existing prognostic tools for the prediction of SSI within gastrointestinal surgery populations.

## Methods

A systematic review was performed according to a predefined protocol (registered on PROSPERO: CRD42022311019) and reported according to the PRISMA guidelines^14^.

### Search strategy and information sources

The search strategy was developed to identify prognostic models that predict the occurrence of SSI after gastrointestinal surgery (*Appendix S1*). A comprehensive search of MEDLINE, Embase, Global Health, and IEEE Xplore was performed on 24 February 2022. This search was supplemented through hand searching citation and reference lists from relevant articles. The searches were limited to publications in the English language due to practical restraints and restricted to the year 2000 onwards to ensure relevance to current surgical practice (unless the model was subsequently validated).

Studies were eligible for inclusion if they developed or externally validated a model that sought to predict risk of SSI after gastrointestinal surgery in adults using preoperative and/or operative characteristics. Models that included patients undergoing non-gastrointestinal surgical procedures were eligible if these also included gastrointestinal surgical procedures. Furthermore, models that were developed before 2000 but externally validated afterwards were eligible. However, the exclusion criteria were: development or validation performed for non-adult patients (less than 18 years) where development and performance were not separate for adults and children; inclusion of postoperative (including administrative data) or context-specific parameters (time interval or individual sites) in the risk model, or the predictors included were not reported; primary outcome was not SSI (that is a composite outcome of postoperative infections or complications); and procedure-specific risk models (for example appendicectomy).

### Study selection and data extraction

After the removal of duplicate publications, titles and abstracts were screened, and full texts of relevant publications uploaded onto the Covidence online systematic review tool^15^ for review against these eligibility criteria. Data fields of interest were extracted from eligible papers, related to study characteristics (year of publication, setting, sample size, inclusion criteria, and techniques for model development), SSI (definition used, number of cases, and method and time frame of follow-up), and the risk model itself (validation status, modelling techniques, clinical parameters included, and any metrics reported regarding prognostic accuracy and model performance). Data extracted were stored on a research electronic data capture (‘REDCap’) server^16^. Study screening and data extraction were completed independently by two among the reviewers (K.A.M., J.S., S.L., T.G., and A.R.), with any disagreements resolved through a consensus-based approach.

### Quality assessment and data synthesis

Quality assessment of eligible studies was performed using ‘Transparent Reporting of a multivariable prediction model for Individual Prognosis Or Diagnosis’ (TRIPOD) reporting guidelines^17^ and the ‘Prediction model Risk Of Bias ASsessment Tool’ (PROBAST)^18^.

A narrative (descriptive) synthesis of results was performed. The data extracted were summarized using frequencies and percentages for dichotomous variables and using medians and interquartile ranges for continuous variables. SSI event rates with 95 per cent confidence intervals were also calculated, where possible. No meta-analysis was planned or performed. Furthermore, the minimum sample size required for developing a multivariable prediction model was calculated for the observed SSI rate and number of candidate predictors evaluated (if reported), and compared with the development-cohort sample size^19^. This was performed irrespective of whether logistic regression or other modelling techniques were applied.

Model performance was compared using the area under the receiver operating characteristic (ROC) curve (AUC) and summarized using the geometric mean and range. Prognostic accuracy summary statistics (sensitivity, specificity, positive predictive value, and negative predictive value) were reported. An AUC of less than 0.6 was considered to indicate ‘poor’ model discrimination, an AUC of 0.6 to less than 0.7 was considered to indicate ‘moderate’ model discrimination, an AUC of 0.7 to less than 0.8 was considered to indicate ‘good’ model discrimination, and an AUC of greater than or equal to 0.8 was considered to indicate ‘excellent’ model discrimination^20^. Models should also be ‘well calibrated’ in be able to accurately predict the outcome of interest across the spectrum of risk—for example, resource wastage or even iatrogenic harm may occur if there is overestimation for patients at low risk and underestimation of patients at high risk. Reporting calibration intercept (calibration-in-the-large) and slope (an intercept of 0 and slope of 1 indicating ‘perfect’ calibration) was considered an appropriate method to assess calibration, in line with current best practice^21^. However, assessment of calibration through a Brier score or Hosmer–Lemeshow test was also extracted. All statistical analyses were performed using RStudio version 4.1.1 (R Foundation for Statistical Computing, Vienna, Austria), with packages including *tidyverse*, *finalfit*, *pmsampsize*^22^, and *predictr*^23^.

## Results

### Search results

**Fig. 1:**
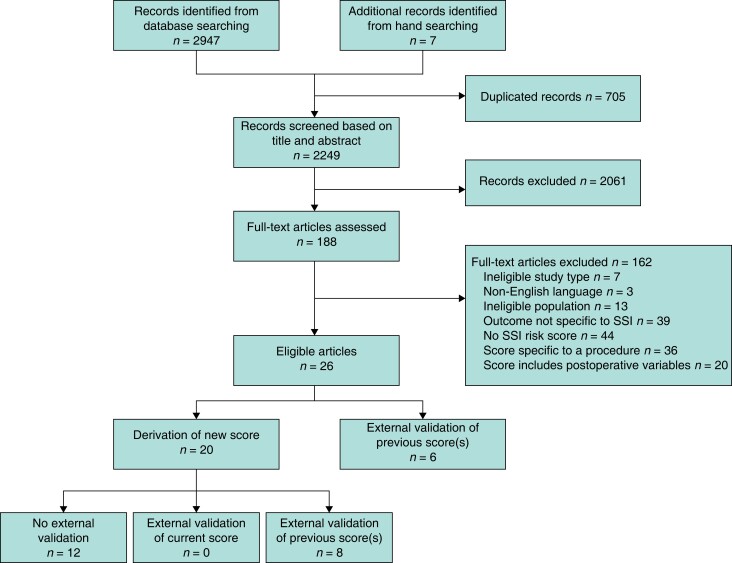
PRISMA flow diagram SSI, surgical-site infection.

In total, 2249 unique records were identified from the literature search, of which 188 (8.4 per cent) full texts were assessed for eligibility (*Fig. 1*). From 26 papers included, there were 23 unique risk models identified that sought to predict the risk of SSI within 30 days after gastrointestinal surgery (*Table 1*). Of these original models, 8 (35 per cent) had score development reported only^12,13,25–27,33,37,44^, 15 (65 per cent) had undergone internal validation^24,28–31,34–36,38–43^, and 4 (17 per cent) had been externally validated^12,13,24,36^ (*Table 1*). The National Nosocomial Infections Surveillance (NNIS) scoring system was most frequently externally validated (67 per cent, 12 of 18), typically being used as a benchmark to compare a novel model.

**Table 1:** Characteristics of included studies describing an original risk score

Score	Country	Study design	Study population	SSI outcome definition	SSI rate	Model approach (variable selection)	Validation
SENIC (1985)^24^	USA	Retrospective, national	Pansurgical	CDC (superficial, deep)	Unreported (total = 117 850)	Logistic regression (forward selection)	Internal (subgroup) and external^25,26^
NNIS (1986)^12^	USA	Retrospective, national	Pansurgical	CDC (full)	2.8% (*n* = 2376/84 691)	Expert opinion (previous model)	External^25–32^
Updated NNIS (2001)^13^	USA	Retrospective, national	General surgery	Undefined	1.0% (*n* = 449/42 815)	Expert opinion (previous model)	External^65^
Smith *et al*. (2004)^33^	USA	Retrospective, single centre	Elective colorectal	CDC (superficial, deep)	25.6% (*n* = 45/176)	Logistic regression (forward selection)	None
de Oliveira *et al*. (2006)^27^	Brazil	Prospective, regional	General surgery	Other (‘purulent secretion’)	24.5% (*n* = 149/608)	Logistic regression (backward selection)	None
Neumayer *et al*. (2007)^28^	USA	Retrospective, national (NSQIP)	General surgery	CDC (superficial, deep)	4.3% (*n* = 7035/163 624)	Logistic regression (forward selection)	Internal (subgroup)
de Campos-Lobato *et al*. (2009)^34^	USA	Retrospective, national (NSQIP)	Colorectal	CDC (organ space)	3.3% (*n* = 728/21 894)	Linear regression (literature)	Internal (subgroup)
Alavi *et al*. (2010)^35^	USA	Retrospective, national (NSQIP)	Colorectal	CDC (full)	23.5% (*n* = 1682/7149)	Logistic regression (backward selection)	Internal (subgroup)
Morales *et al*. (2011)^25^	Canada	Prospective, single centre	Emergency general surgery	CDC (deep, organ space)	13.8% (*n* = 85/614)	Logistic regression (forward selection)	None
RSSIC (2012)^29^	USA	Prospective, single centre	Elective general surgery	CDC (full)	24.3% (*n* = 122/503)	Logistic regression (univariable threshold)	Internal (bootstrap)
COLA (2012)^36^	Switzerland	Prospective, regional	Colorectal	CDC (full)	21.3% (*n* = 114/534)	Logistic regression (forward selection)	Internal (cross-validation) and external^37^
SSIRS (2013)^30^	USA	Retrospective, national (NSQIP)	Pansurgical	CDC (full)	3.9% (*n* = 14 227/363 040)	Logistic regression (forward selection)	Internal (subgroup)
Hedrick *et al*. (2013)^38^	USA	Retrospective, national (NSQIP)	Elective colorectal	CDC (superficial, deep)	9.3% (*n* = 1719/18 403)	Logistic regression (backward selection)	Internal (bootstrap)
SSISECC (2018)^26^	China	Retrospective, single centre	Elective colorectal	Undefined	7.1% (*n* = 72/1008)	Logistic regression (univariable threshold)	None
Grant *et al*. (2019)^37^	Switzerland, France, UK	Retrospective, international	Colorectal	CDC (full)	Unreported (total = 46 320)	Logistic regression (forward selection)	None
PREVENTT (2019)^39^	USA	Retrospective, regional (NSQIP)	Colorectal	Undefined	21.1% (*n* = 366/1737)	Logistic regression (backward selection)	Internal (bootstrap)
Kocbek *et al*. (2019)^40^	Norway	Retrospective, single centre	Colorectal	Other (ICD-10, NCSP codes)	20.5% (*n* = 233/1137)	Machine learning (XGBoost)	Internal (cross-validation)
Wei *et al*. (2019)^41^	USA	Prospective, single centre	Emergency General surgery	CDC (organ space)	14.7% (*n* = 172/1171)	Logistic regression (Bayesian regularisation)	Internal (subgroup)
Angel García *et al*. (2020)^42^	Spain	Retrospective, regional	Colorectal	Undefined	7.3% (*n* = 463/6325)	Logistic regression (backward selection)	Internal (bootstrap)
AMRAMS (2020)^31^	China	Retrospective, single centre	Multiple (general surgery, gynaecology, orthopaedics, urology)	Undefined	1.1% (*n* = 244/21 611)	Machine learning (LASSO)	Internal (subgroup)
						Machine learning (CNN)	Internal (subgroup)
Boubekki *et al*. (2021)^43^	Norway	Retrospective, single centre	Colorectal	Other (ICD-10, NCSP codes)	20.5% (*n* = 233/1137)	Machine learning (GBOOST)	Internal (cross-validation)
Fernandez-Moure *et al*. (2021)^44^	USA	Retrospective, single centre	Emergency general surgery	Other (ICD-9 codes)	13.3% (*n* = 632/4738)	Logistic regression (expert opinion)	None

CDC, Centers for Disease Control and Prevention; NSQIP, National Surgical Quality Improvement Program; NCSP, NOMESCO Classification of Surgical Procedures; LASSO, least absolute shrinkage and selection operator; CNN, convolutional neural network.

### Characteristics of included studies

The models were typically based on single-centre studies (44 per cent, 10 of 23), with no models developed using prospective national or international data. Furthermore, of the nine studies based on national or international data, 56 per cent (five of nine) were based on the same US-based registry (National Surgical Quality Improvement Program (‘NSQIP’)). Overall, almost all models (83 per cent, 19 of 23) were developed using data from high-income countries, with the remainder from upper-middle-income countries (Brazil, China). There was also no patient–public involvement identified across included models.

Furthermore, there were important differences in the underlying populations included, with most involving patients either undergoing colorectal (48 per cent, 11 of 23) or general surgical (30 per cent, 7 of 23) procedures, with the others also including some or all other surgical specialties (22 per cent, 5 of 23). Furthermore, whereas most models included all procedures irrespective of operative urgency, a minority included only elective (17 per cent, 4 of 23) or emergency (13 per cent, 3 of 23) procedures.

Within the included studies, SSI was typically defined according to the Centers for Disease Control and Prevention (CDC) criteria (57 per cent, 13 of 23), with the rest providing no clear definition (26 per cent, 6 of 23) or alternative definitions based on administrative codes or other clinical signs (17per cent, 4 of 23) (*Table 1*). However, even among studies using the CDC criteria, only a minority used the full definition (46 per cent, 6 of 13), with the rest including a combination of superficial, deep, or organ-space SSI.

**Fig. 2:**
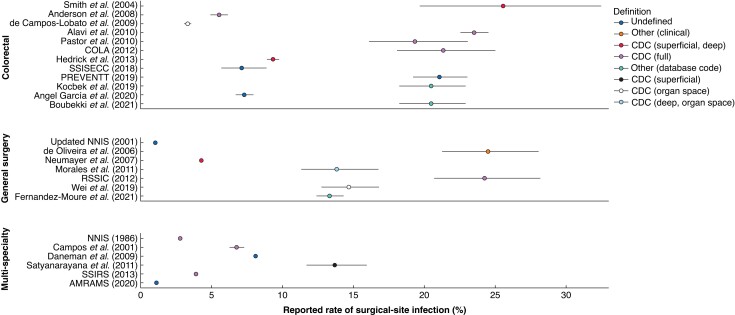
Rate of 30-day surgical site infection outcome reported in included studies, by definition used. Where not reported, 95% binomial confidence intervals were calculated. CDC, Centers for Disease Control and Prevention.

Overall, there was a wide variation in the SSI rates reported between studies (1.0 per cent^13^ to 25.6 per cent^33^), with the highest rates observed in colorectal populations and the lowest rates observed in multi-specialty populations (*Fig. 2*). However, there was no clear pattern in the SSI rate observed according to the definition used.

### Variable selection

Predictive factors were predominantly identified via logistic regression (74 per cent, 17 of 23) (*Table 1*), with a minority involving novel machine-learning-based approaches (17 per cent, 4 of 23). However, there were substantial methodological concerns with most variable selection approaches used, with a majority conducting selection based on: stepwise approaches (52 per cent, 12 of 23), univariable significance (9 per cent, 2 of 23), or expert opinion alone (13 per cent, 3 of 23). Where reported, only 42.9 per cent (9 of 21) of derivation cohorts achieved the minimum sample size required to develop a predictive model based on the SSI rate reported and the number of candidate predictors being explored (*Fig. S1*).

**Fig. 3:**
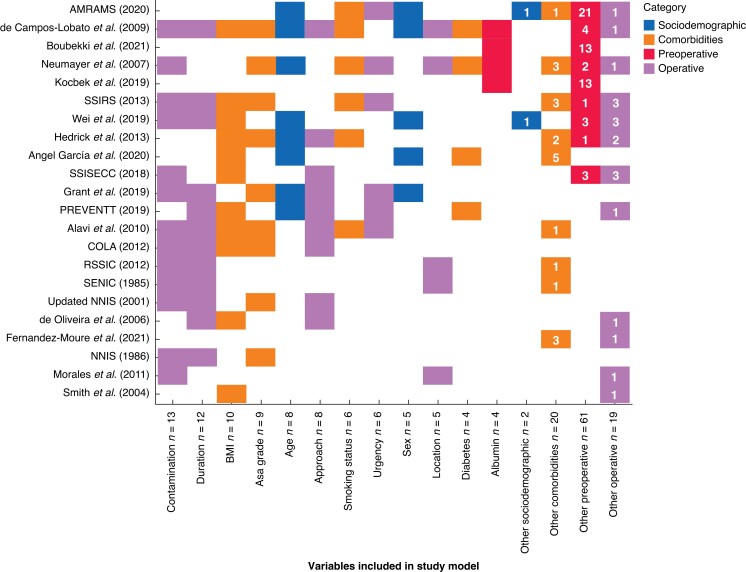
Tile plot of variables included in all models identified on systematic review. Numbers on tiles represent the number of variables in the “other” categories within each model (variables included in <2 models) - see *Table S2* for full list.

There was substantial heterogeneity in the number and examples of predictors identified across models (median 7, range 2–28). However, commonly identified predictors involved operative and patient-specific factors, with most models highlighting the importance of operative contamination (57 per cent, 13 of 23) and duration (52 per cent, 12 of 23) (*Fig. 3* and *Table S1*). Overall, three models were solely formed of predictors available before operation, whereas the majority of models also included predictors requiring intraoperative information (87 per cent, 20 of 23).

### Quality assessment of included studies

Adherence to the TRIPOD reporting guidelines was mixed, with consistently poor reporting for methods of blinding to outcome or predictors, how missing data were handled, and reporting of model results and evaluation (*Fig. S2*). All models demonstrated a high risk of bias, principally due to the analytic approach and outcome definition, and none displayed high applicability to an undifferentiated gastrointestinal surgical population (*Fig. S3*).

### Model discrimination and prognostic accuracy

**Fig. 4:**
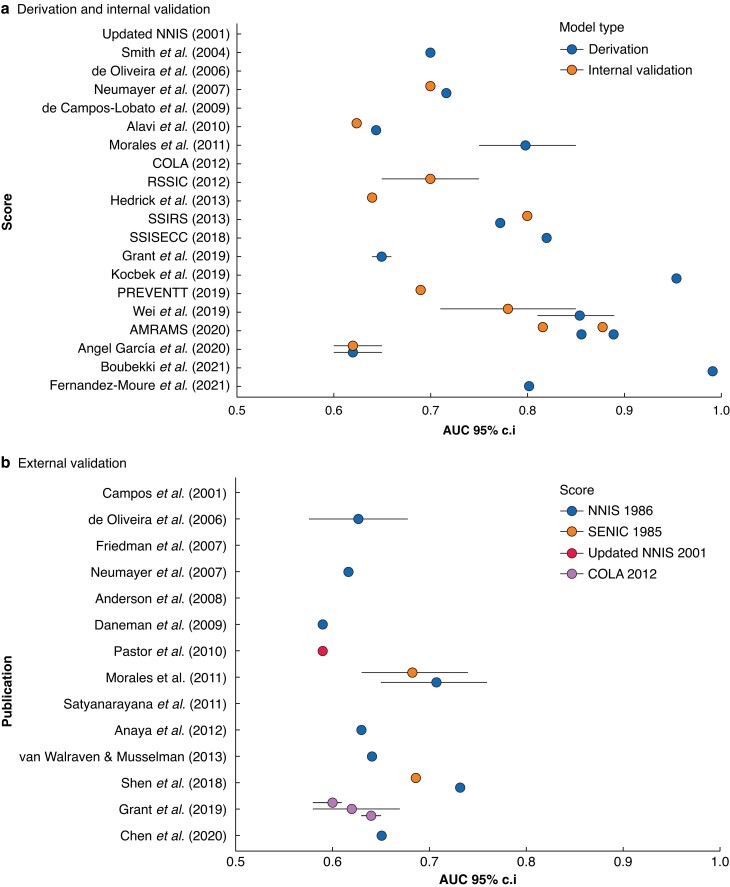
Discriminatory performance of all prognostic models to predict surgical site infection in the 30-day postoperative period. Where not reported, no AUC is displayed.

Of the 23 unique models identified, discrimination was reported in 61 per cent (14 of 23) for the derivation cohort, with the majority reporting ‘good’ or ‘excellent’ discrimination (79 per cent (11 of 14), AUC geometric mean 0.831, AUC range 0.620–0.991) (*Fig. 4a* and *Table S2*). Of the 15 studies that subsequently conducted internal validation, 67 per cent (10 of 15) reported discrimination. There was a reduction in models reporting ‘good’ or ‘excellent’ discrimination when internal validation was performed (60 per cent (6 of 10), AUC geometric mean 0.735, AUC range 0.620–0.878).

In comparison, of the four models that underwent external validation, the AUC remained greater than or equal to 0.7 for one model (the NNIS model) in 17 per cent (2 of 12) of cohorts (*Fig. 4b* and *Table S2*). However, there was no evidence to confirm this as statistically significant ‘good’ discrimination for SSI with an AUC greater than or equal to 0.7.

However, only a minority of models assessed model calibration (22 per cent, 5 of 23) or prognostic accuracy (17 per cent, 4 of 23) during development or internal validation (*Table S2*). These were less frequently reported in external validation studies (prognostic accuracy 6 per cent (1 of 23), calibration 11 per cent (2 of 23)). Even when reported, calibration was insufficiently assessed, with only one model reporting the calibration intercept and slope in line with current best practice.

## Discussion

This systematic review identified 23 original models developed for the prediction of SSI that were relevant to patients undergoing gastrointestinal surgery. Like many predictive models developed for other outcomes of interest^10^, significant concerns have been identified around methodological quality, evaluation, and clinical relevance to an undifferentiated gastrointestinal surgical population. Furthermore, even among the four (17 per cent) models that had undergone external validation^12,13,24,36^, none significantly exceeded the a priori threshold for ‘good’ discrimination (AUC greater than or equal to 0.7) and almost all did not report assessment of calibration or prognostic accuracy.

Predicting patients at high risk of SSI would facilitate shared perioperative decision-making and allow targeting of resources to those most likely to benefit. Yet, despite over 30 years of research into the prediction of SSI, this remains a challenging task for several reasons^45^. SSI is inherently multifactorial, with numerous risk factors previously established^46^, encompassing an interplay of patient, operative, and hospital-based determinants. This leads to many candidate predictors, increasing the minimum sample size required, meaning development becomes limited to data from large-scale registries or prospective studies. A breadth of prognostic factors were identified in this systematic review—both in number and type across different models. While the most frequently identified prognostic factors likely represent those of greatest relevance for prediction of SSI, these should be interpreted with caution given the highlighted methodological concerns and heterogeneity in underlying populations. Future risk-prediction models may seek to prioritize investigation of these factors, although they will need to consider the potential for confounders, statistical error, and/or collinearity if not accounted for in the original models. Furthermore, these should use robust modelling approaches for variable selection (penalized regression or machine-learning approaches, rather than univariable selection or stepwise regression) or internal validation (bootstrapping or cross-validation, over random data-set splitting)^18^. While the CDC definition of SSI represents an established gold-standard definition, this is inherently subjective and requires in-person assessment^47^. Particularly with the adoption of enhanced recovery after surgery (‘ERAS’) programmes, SSI increasingly occurs after hospital discharge^48^. Therefore, studies conducted retrospectively that lack robust follow-up, or in areas of poor healthcare access, may underestimate the true event rate. These concerns were seen in many of the models identified, which may partly explain poor prediction in those externally validated. However, even with an established gold standard, there remained heterogeneity in the outcome of interest given that a significant minority of models used a non-CDC outcome or considered only specific subtypes of SSI. This limits the comparability between models and signals a lack of consensus on what aspect(s) of SSI should be the intended predictive target. Finally, the sample size and study design of included studies often did not meet required expectations. Only a minority of models were based on estimates of a minimum sample size, all of which were based on retrospective data (*Fig. S1* and *Table 1*). As multicentre prospective studies may be expensive and/or complex to conduct, retrospective data should only be used when there are sufficient event rates, data quality, and modelling approaches to account for inherent biases^18^.

Assessment of model performance was limited by the overall poor quality of reporting (particularly prognostic accuracy and model calibration), as well as the high risk of bias and scarce external validation. Overall, most models performed well with regard to derivation and internal validation, with the highest discrimination observed in models based on machine learning^31,43^. These are increasingly common in the literature^49^, with 30 per cent (3 of 10) of SSI models developed in the last 5 years using machine-learning approaches. Although at an early stage within healthcare, these machine-learning-based models have theoretical benefits, including better handling of non-linearity and the incorporation of interaction terms, with the potential to improve predictive accuracy^50^. However, these can require significantly more data to achieve stability, are prone to overfitting, are less transparent for patients and clinicians, and often do not provide clinically significant enhancement to discrimination over well-conducted regression approaches^51,52^.

While several models appear promising, confirmation using external validation is essential before these can be trusted or used in clinical practice^11^. Despite this, there continues to be difficulties with reproducibility across the broader prediction literature^53^. This is reflected here, with only one in six having undergone external validation. This may be due in part to the quantity, heterogeneity, and complexity of variables identified in models, posing practical challenges to validation and clinical usage if these data are not routinely recorded or available^54,55^. Even when this was performed, as expected there was a reduction in the observed discrimination compared with the derivation cohorts^53^. Of all models, the NNIS model remains the most validated model in the literature, likely in part due to its simplicity, being among the first published^13^, and its use in risk adjustment to allow inter-centre comparison of SSI rates^7^. While it did not demonstrate ‘good’ discrimination on external validation, it still displayed the highest discrimination reported. Nevertheless, particularly as the prognostic accuracy and calibration are unclear, the clinical utility remains low. Furthermore, it should be noted that almost all model derivation and validation has occurred in the context of high-income countries. It remains unclear whether any models can be generalized to low- and middle-income countries, which continue to experience the greatest burden of SSI^1^. Additional external validation or development of models relevant to these contexts are needed to ensure equitable benefit.

This systematic review has several key strengths. It has comprehensively identified and evaluated predictive models previously developed for SSI after gastrointestinal surgery. Each model has been compared with current best practice regarding the reporting quality, risk of bias, minimum sample size, and practice of external validation—this allows a clear understanding of the suitability for the original purpose intended, as well as for prediction in undifferentiated populations of patients undergoing gastrointestinal surgery. This also provides a clear framework of standards for future models to meet. However, there are several important limitations to this study. First, the search was limited to English-language papers and databases, and so the systematic review may not encompass every possible model developed globally. Second, only models that were not procedure specific were included. While these may share common prognostic factors relevant to broader populations, it was anticipated this would be limited due to procedure-specific variables and poor performance when transported to a broader population^56^.

Prognostic models should be deliverable within the clinical context, have a clear target population with utility within the clinical decision process, and be demonstrated to be acceptable to patients and clinicians. Across the wider predictive-model literature, there is a gulf between the number of models developed and those adopted into routine practice^57^. No models have been recommended for individual risk assessment of SSI within guidelines or routinely adopted on a large-scale basis^7,58,59^. Indeed, there is limited evidence to support the use of any in undifferentiated gastrointestinal surgical patients (and even within the original subgroups of interest). Therefore, ongoing work to address this gap in prognostic models validated for a global gastrointestinal surgical population is underway by the National Institute for Health and Care Research (‘NIHR') Global Health Research Unit on Global Surgery. There are numerous evidence-based interventions already available before, during, and after surgery that modify the risk of SSI and minimize associated harm^7,58,59^. Yet, without an adequate understanding of how to stratify patients according to their risk, shared decision-making and the appropriate allocation of targeted enhanced monitoring and perioperative interventions for SSI remain challenging^32^. Further, comprehensive external validation of existing models or novel, validated prognostic tools are needed to better differentiate risk of SSI across a global population of gastrointestinal surgery patients.

## Supplementary Material

znad187_Supplementary_Data

## Data Availability

The data sets generated during and/or analysed during the current study are available from the corresponding author on reasonable request.

## References

[1] GlobalSurg Collaborative . Surgical site infection after gastrointestinal surgery in high-income, middle-income, and low-income countries: a prospective, international, multicentre cohort study. Lancet Infect Dis 2018;18:516–525 29452941 10.1016/S1473-3099(18)30101-4 PMC5910057

[2] National Institute for Health Research Global Research Health Unit on Global Surgery . Delphi prioritization and development of global surgery guidelines for the prevention of surgical-site infection. Br J Surg 2020;107:970–977 32212158 10.1002/bjs.11530 PMC7317442

[3] Gelhorn HL , Anand SB, Parvizi J, Morrison T, Yu H, Pokrzywinski R et al Qualitative interviews to identify burden of illness, impacts and costs associated with surgical site infections. J Comp Eff Res 2017;7:357–367 29120226 10.2217/cer-2017-0075 PMC6615409

[4] Allegranzi B , Bagheri Nejad S, Combescure C, Graafmans W, Attar H, Donaldson L et al Burden of endemic health-care-associated infection in developing countries: systematic review and meta-analysis. Lancet 2011;377:228–241 21146207 10.1016/S0140-6736(10)61458-4

[5] Pinkney TC , Bartlett M, Gheorghe DC, Redman A, Dowswell V, Hawkins G et al Impact of wound edge protection devices on surgical site infection after laparotomy: multicentre randomised controlled trial (ROSSINI trial). BMJ 2013;347:f4305 10.1136/bmj.f4305 PMC3805488 23903454

[6] Monahan M , Jowett S, Pinkney T, Brocklehurst P, Morton DG, Abdali Z et al Surgical site infection and costs in low- and middle-income countries: a systematic review of the economic burden. PLoS One 2020;15:e0232960 10.1371/journal.pone.0232960 PMC7272045 32497086

[7] WHO . Global Guidelines for the Prevention of Surgical Site Infection. Geneva: WHO, 2018 30689333

[8] Temple R . Enrichment of clinical study populations. Clin Pharmacol Ther 2010;88:774–778 20944560 10.1038/clpt.2010.233

[9] Sangsuwan T , Jamulitrat S, Watcharasin P. Risk adjustment performance between NNIS index and NHSN model for postoperative colorectal surgical site infection: a retrospective cohort study. Ann Med Surg 2022;77:103715 10.1016/j.amsu.2022.103715 PMC9142714 35637982

[10] Bouwmeester W , Zuithoff NPA, Mallett S, Geerlings MI, Vergouwe Y, Steyerberg EW et al Reporting and methods in clinical prediction research: a systematic review. PLoS Med 2012;9:e1001221 10.1371/journal.pmed.1001221 PMC3358324 22629234

[11] Collins GS , de Groot JA, Dutton S, Omar O, Shanyinde M, Tajar A et al External validation of multivariable prediction models: a systematic review of methodological conduct and reporting. BMC Med Res Methodol 2014;14:40 24645774 10.1186/1471-2288-14-40 PMC3999945

[12] Culver DH , Horan TC, Gaynes RP, Martone WJ, Jarvis WR, Emori TG et al Surgical wound infection rates by wound class, operative procedure, and patient risk index. National Nosocomial Infections Surveillance System. Am J Med 1991;91:152S –157S 1656747 10.1016/0002-9343(91)90361-z

[13] Gaynes RP , Culver DH, Horan TC, Edwards JR, Richards C, Tolson JS et al Surgical site infection (SSI) rates in the United States, 1992–1998: the National Nosocomial Infections Surveillance System basic SSI risk index. Clin Infect Dis 2001; 33(Suppl 2): S69–S77 11486302 10.1086/321860

[14] Moher D , Liberati A, Tetzlaff J, Altman DG. Preferred reporting items for systematic reviews and meta-analyses: the PRISMA statement. BMJ 2009;339:b2535 PMC3090117 21603045

[15] Cochrane Community . *Covidence*. https://community.cochrane.org/help/tools-and-software/covidence (accessed 1 March 2023)

[16] Harris PA , Taylor R, Thielke R, Payne J, Gonzalez N, Conde JG. Research electronic data capture (REDCap)—a metadata-driven methodology and workflow process for providing translational research informatics support. J Biomed Inform 2009;42:377–381 18929686 10.1016/j.jbi.2008.08.010 PMC2700030

[17] Collins GS , Reitsma JB, Altman DG, Moons KGM. Transparent reporting of a multivariable prediction model for individual prognosis or diagnosis (TRIPOD): the TRIPOD statement. BMC Med 2015;13:1 25563062 10.1186/s12916-014-0241-z PMC4284921

[18] Wolff RF , Moons KGM, Riley RD, Whiting PF, Westwood M, Collins GS et al PROBAST: a tool to assess the risk of bias and applicability of prediction model studies. Ann Intern Med 2019;170:51–58 30596875 10.7326/M18-1376

[19] Riley RD , Snell KIE, Ensor J, Burke DL, Harrell FE Jr, Moons KGM et al Minimum sample size for developing a multivariable prediction model: PART II - binary and time-to-event outcomes. Stat Med 2019;38:1276–1296 30357870 10.1002/sim.7992 PMC6519266

[20] Fischer JE , Bachmann LM, Jaeschke R. A readers’ guide to the interpretation of diagnostic test properties: clinical example of sepsis. Intensive Care Med 2003;29:1043–1051 12734652 10.1007/s00134-003-1761-8

[21] Van Calster B , McLernon DJ, van Smeden M, Wynants L, Steyerberg EW, Bossuyt P et al Calibration: the Achilles heel of predictive analytics. BMC Med 2019;17:230 31842878 10.1186/s12916-019-1466-7 PMC6912996

[22] Ensor J , Martin EC, Riley RD. *pmsampsize: Calculates the Minimum Sample Size Required for Developing a Multivariable Prediction Model*. https://cran.r-project.org/web/packages/pmsampsize (accessed 1 March 2023)

[23] McLean K , Knight SR, Harrison EM. *predictr: An Integrated Workflow to Develop, Evaluate, and Output Predictive Models in R*. https://github.com/kamclean/predictr (accessed 1 March 2023)

[24] Haley RW , Culver DH, Morgan WM, White JW, Emori TG, Hooton TM. Identifying patients at high risk of surgical wound infection. A simple multivariate index of patient susceptibility and wound contamination. Am J Epidemiol 1985;121:206–215 4014116 10.1093/oxfordjournals.aje.a113991

[25] Morales CH , Escobar RM, Villegas MI, Castano A, Trujillo J. Surgical site infection in abdominal trauma patients: risk prediction and performance of the NNIS and SENIC indexes. Can J Surg 2011;54:17–24 21251428 10.1503/cjs.022109 PMC3038362

[26] Shen Z , Lin Y, Ye Y, Jiang K, Xie Q, Gao Z et al The development and validation of a novel model for predicting surgical complications in colorectal cancer of elderly patients: results from 1008 cases. Euro J Surg Oncol 2018;44:490–495 10.1016/j.ejso.2018.01.007 29402555

[27] de Oliveira AC , Ciosak SI, Ferraz EM, Grinbaum RS. Surgical site infection in patients submitted to digestive surgery: risk prediction and the NNIS risk index. Am J Infect Control 2006;34:201–207 16679177 10.1016/j.ajic.2005.12.011

[28] Neumayer L , Hosokawa P, Itani K, El-Tamer M, Henderson WG, Khuri SF. Multivariable predictors of postoperative surgical site infection after general and vascular surgery: results from the patient safety in surgery study. J Am Coll Surg 2007;204:1178–1187 17544076 10.1016/j.jamcollsurg.2007.03.022

[29] Anaya DA , Cormier JN, Xing Y, Koller P, Gaido L, Hadfield D et al Development and validation of a novel stratification tool for identifying cancer patients at increased risk of surgical site infection. Ann Surg 2012;255:134–139 22143206 10.1097/SLA.0b013e31823dc107

[30] van Walraven C , Musselman R. The surgical site infection risk score (SSIRS): a model to predict the risk of surgical site infections. PLoS One 2013;8:e67167 10.1371/journal.pone.0067167 PMC3694979 23826224

[31] Chen W , Lu Z, You L, Zhou L, Xu J, Chen K. Artificial intelligence-based multimodal risk assessment model for surgical site infection (AMRAMS): development and validation study. JMIR Med Inform 2020;8:e18186 10.2196/18186 PMC7325005 32538798

[32] Odor PM , Bampoe S, Gilhooly D, Creagh-Brown B, Moonesinghe SR. Perioperative interventions for prevention of postoperative pulmonary complications: systematic review and meta-analysis. BMJ 2020;368:m540 10.1136/bmj.m540 PMC7190038 32161042

[33] Smith RL , Bohl JK, McElearney ST, Friel CM, Barclay MM, Sawyer RG et al Wound infection after elective colorectal resection. Ann Surg 2004;239:599–607 15082963 10.1097/01.sla.0000124292.21605.99 PMC1356267

[34] de Campos-Lobato LF , Wells B, Wick E, Pronty K, Kiran R, Remzi F et al Predicting organ space surgical site infection with a nomogram. J Gastrointest Surg 2009;13:1986–1992 19760301 10.1007/s11605-009-0968-6

[35] Alavi K , Sturrock PR, Sweeney WB, Maykel JA, Cervera-Servin JA, Tseng J et al A simple risk score for predicting surgical site infections in inflammatory bowel disease. Dis Colon Rectum 2010;53:1480–1486 20940595 10.1007/DCR.0b013e3181f1f0fd

[36] Gervaz P , Bandiera-Clerc C, Buchs NC, Eisenring MC, Troillet N, Perneger T et al Scoring system to predict the risk of surgical-site infection after colorectal resection. Br J Surg 2012;99:589–595 22231649 10.1002/bjs.8656

[37] Grant R , Aupee M, Buchs NC, Cooper K, Eisenring M-C, Lamagni T et al Performance of surgical site infection risk prediction models in colorectal surgery: external validity assessment from three European national surveillance networks. Infect Control Hosp Epidemiol 2019;40:983–990 31218977 10.1017/ice.2019.163

[38] Hedrick TL , Sawyer RG, Friel CM, Stukenborg GJ. A method for estimating the risk of surgical site infection in patients with abdominal colorectal procedures. Dis Colon Rectum 2013;56:627–637 23575403 10.1097/DCR.0b013e318279a93e

[39] Bordeianou L , Cauley CE, Patel R, Bleday R, Mahmood S, Kennedy K et al Prospective creation and validation of the PREVENTT (prediction and enaction of prevention treatments trigger) scale for surgical site infections (SSIs) in patients with diverticulitis. Ann Surg 2019;270:1124–1130 29916880 10.1097/SLA.0000000000002859

[40] Kocbek P , Fijacko N, Soguero-Ruiz C, Mikalsen KO, Maver U, Povalej Brzan P et al Maximizing interpretability and cost-effectiveness of surgical site infection (SSI) predictive models using feature-specific regularized logistic regression on preoperative temporal data. Comput Math Methods Med 2019;2019:2059851 10.1155/2019/2059851 PMC6399553 30915154

[41] Wei S , Green C, Kao LS, Padilla-Jones BB, Truong VTT, Wade CE et al Accurate risk stratification for development of organ/space surgical site infections after emergent trauma laparotomy. J Trauma Acute Care Surg 2019;86:226–231 30531329 10.1097/TA.0000000000002143 PMC7004798

[42] Angel García D , Martinez Nicolas I, Garcia Marin JA, Soria Aledo V. Risk-adjustment models for clean and colorectal surgery surgical site infection for the Spanish health system. Int J Qual Health Care 2020;32:599–608 32901796 10.1093/intqhc/mzaa104

[43] Boubekki A , Nordhaugmyhre J, Luppino LT, Mikalsen K, Revhaug A, Jenssen R. Clinically relevant features for predicting the severity of surgical site infections. IEEE J Biomed Health Inform 2021;26:1794–1801 10.1109/JBHI.2021.3121038 34665748

[44] Fernandez-Moure JS , Wes A, Kaplan LJ, Fischer JP. Actionable risk model for the development of surgical site infection after emergency surgery. Surg Infect (Larchmt) 2021;22:168–173 32397903 10.1089/sur.2019.282

[45] Shah N , Hamilton M. Clinical review: can we predict which patients are at risk of complications following surgery? Crit Care 2013;17:226 23672931 10.1186/cc11904 PMC3672530

[46] Korol E , Johnston K, Waser N, Sifakis F, Jafri HS, Lo M et al A systematic review of risk factors associated with surgical site infections among surgical patients. PLoS One 2013;8:e83743 10.1371/journal.pone.0083743 PMC3867498 24367612

[47] Centers for Disease Control and Prevention (CDC) National Healthcare Safety Network (NHSN) . Patient Safety Component (PSC) Manual Chapter 9: Surgical Site Infection (SSI) Event. Atlanta: CDC, 2016

[48] Woelber E , Schrick EJ, Gessner BD, Evans HL. Proportion of surgical site infections occurring after hospital discharge: a systematic review. Surg Infect 2016;17:510–519 10.1089/sur.2015.241 27463235

[49] Dhiman P , Ma J, Andaur Navarro CL, Speich B, Bullock G, Damen JAA et al Methodological conduct of prognostic prediction models developed using machine learning in oncology: a systematic review. BMC Med Res Methodol 2022;22:101 35395724 10.1186/s12874-022-01577-x PMC8991704

[50] Davenport T , Kalakota R. The potential for artificial intelligence in healthcare. Future Healthc J 2019;6:94–98 10.7861/futurehosp.6-2-94 PMC6616181 31363513

[51] van der Ploeg T , Austin PC, Steyerberg EW. Modern modelling techniques are data hungry: a simulation study for predicting dichotomous endpoints. BMC Med Res Methodol 2014;14:137 25532820 10.1186/1471-2288-14-137 PMC4289553

[52] Andaur Navarro CL , Damen JAA, Takada T, Nijman SWJ, Dhiman P, Ma J et al Risk of bias in studies on prediction models developed using supervised machine learning techniques: systematic review. BMJ 2021;375:n2281 10.1136/bmj.n2281 PMC8527348 34670780

[53] Siontis GC , Tzoulaki I, Castaldi PJ, Ioannidis JP. External validation of new risk prediction models is infrequent and reveals worse prognostic discrimination. J Clin Epidemiol 2015;68:25–34 25441703 10.1016/j.jclinepi.2014.09.007

[54] Wynants L , Collins GS, Van Calster B. Key steps and common pitfalls in developing and validating risk models. BJOG 2017;124:423–432 27362778 10.1111/1471-0528.14170

[55] Chu KM , Weiser TG. Real-world implementation challenges in low-resource settings. Lancet Glob Health 2021;9:e1341–e1342 34418381 10.1016/S2214-109X(21)00310-7

[56] Justice AC , Covinsky KE, Berlin JA. Assessing the generalizability of prognostic information. Ann Intern Med 1999;130:515–524 10075620 10.7326/0003-4819-130-6-199903160-00016

[57] van Royen FS , Moons KGM, Geersing G-J, van Smeden M. Developing, validating, updating and judging the impact of prognostic models for respiratory diseases. Euro Respir J 2022;60:2200250 10.1183/13993003.00250-2022 35728976

[58] Berríos-Torres SI , Umscheid CA, Bratzler DW, Leas B, Stone EC, Kelz RR et al Centers for Disease Control and Prevention guideline for the prevention of surgical site infection, 2017. JAMA Surg 2017;152:784–791 28467526 10.1001/jamasurg.2017.0904

[59] National Institute for Health and Clinical Excellence (NICE) . NICE Guideline [NG125]: Surgical Site Infections: Prevention and Treatment. London: NICE, 2020 31211539

