# Supplementary material for: Prognostic models for surgical-site infection in gastrointestinal surgery: systematic review
Source: Br J Surg. 2023 Jul 12;110(11):1441–50. doi: 10.1093/bjs/znad187 (PMC10564404; doi:10.1093/bjs/znad187)
Supplement: znad187_Supplementary_Data [file znad187_supplementary_data.docx]

**Supplementary Materials - Index**

[**Supplementary Appendixes** 3](file:///D:\Kenneth\Work\2.%20PhD\PhD%20Thesis\2.%20Section%202%20-%20Implementation\2.%20Target%20Population\1.%20SSI%20Review\BJS\R1\4_ssi_review_r1_supplement.docx#_Toc131168496)

[Appendix 1: Search strategy for published risk prediction models. 3](file:///D:\Kenneth\Work\2.%20PhD\PhD%20Thesis\2.%20Section%202%20-%20Implementation\2.%20Target%20Population\1.%20SSI%20Review\BJS\R1\4_ssi_review_r1_supplement.docx#_Toc131168497)

[**Supplementary Figures and Tables** 4](file:///D:\Kenneth\Work\2.%20PhD\PhD%20Thesis\2.%20Section%202%20-%20Implementation\2.%20Target%20Population\1.%20SSI%20Review\BJS\R1\4_ssi_review_r1_supplement.docx#_Toc131168498)

[Supplementary Figure 1: Evaluation of minimal sample size threshold achievement for score derivation. Black circles represent the minimum sample size required to be achieved, coloured circles represent actual sample size achieved (green = minimum sample size met, red = minimum sample size not met). 4](file:///D:\Kenneth\Work\2.%20PhD\PhD%20Thesis\2.%20Section%202%20-%20Implementation\2.%20Target%20Population\1.%20SSI%20Review\BJS\R1\4_ssi_review_r1_supplement.docx#_Toc131168499)

[Supplementary Figure 2: Adherence to TRIPOD reporting guidelines ^17^ for included studies 5](file:///D:\Kenneth\Work\2.%20PhD\PhD%20Thesis\2.%20Section%202%20-%20Implementation\2.%20Target%20Population\1.%20SSI%20Review\BJS\R1\4_ssi_review_r1_supplement.docx#_Toc131168500)

[Supplementary Figure 3: Adherence to PROBAST ^18^ quality assessment for included studies 6](file:///D:\Kenneth\Work\2.%20PhD\PhD%20Thesis\2.%20Section%202%20-%20Implementation\2.%20Target%20Population\1.%20SSI%20Review\BJS\R1\4_ssi_review_r1_supplement.docx#_Toc131168501)

[Supplementary Table 1: Full specification of additional variables included in all original models identified on systematic review. 7](file:///D:\Kenneth\Work\2.%20PhD\PhD%20Thesis\2.%20Section%202%20-%20Implementation\2.%20Target%20Population\1.%20SSI%20Review\BJS\R1\4_ssi_review_r1_supplement.docx#_Toc131168502)

[Supplementary Table 2: Full specification of model evaluation metrics across derivation and validation cohorts. 8](file:///D:\Kenneth\Work\2.%20PhD\PhD%20Thesis\2.%20Section%202%20-%20Implementation\2.%20Target%20Population\1.%20SSI%20Review\BJS\R1\4_ssi_review_r1_supplement.docx#_Toc131168503)

# **Supplementary Appendixes**

## *Appendix 1: Search strategy for published risk prediction models.*

**Medline / EMBASE / Global Health**

| 1. **Surgical site infection** | surgical?site infection OR (“wound infection” and (surgery or surgical)) |
| --- | --- |
| 1. **Prognostic** | (prognosis or predict* or risk) |
| 1. **Score** | (develop* or deriv* or validat*) and (score or scoring or regression or nomogram or risk index) |

**IEEE Xplore**

| 1. Surgical site infection | surgical?site infection or (wound infection and (surgery or surgical)) |
| --- | --- |

# **Supplementary Figures and Tables**

## *Supplementary Figure 1: Evaluation of minimal sample size threshold achievement for score derivation. Black circles represent the minimum sample size required to be achieved, coloured circles represent actual sample size achieved (green = minimum sample size met, red = minimum sample size not met).*

*
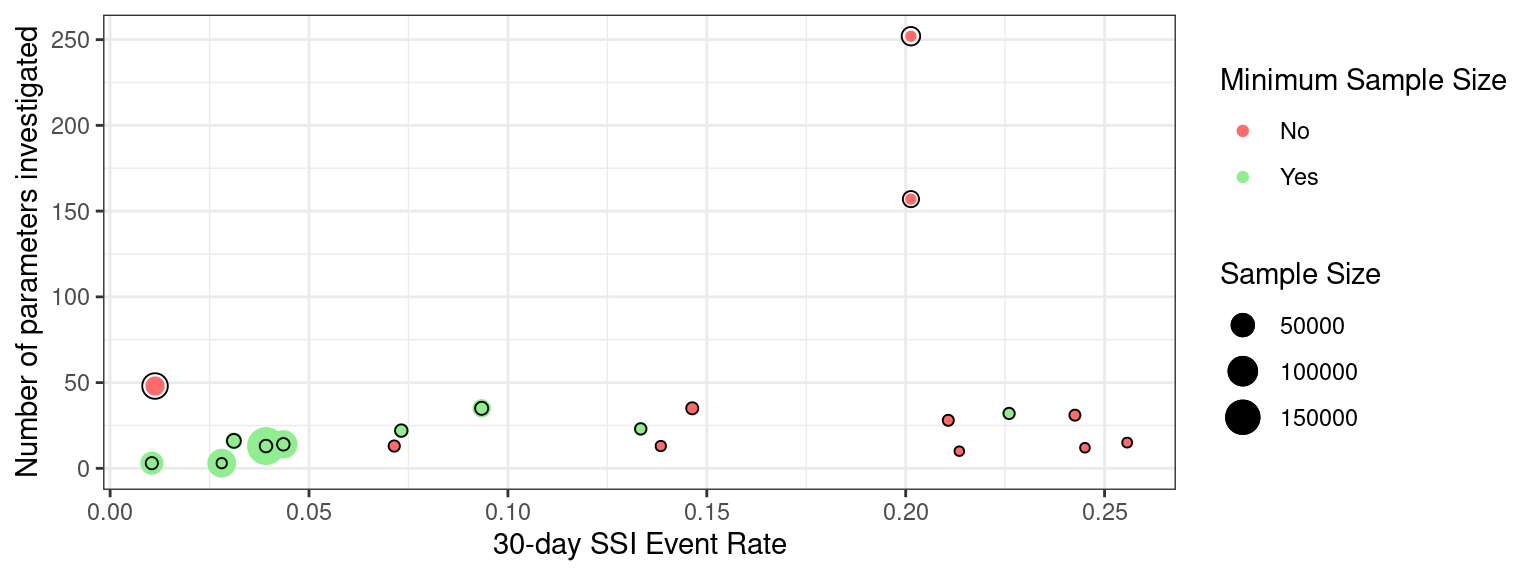
*

## *Supplementary Figure 2: Adherence to TRIPOD reporting guidelines* ^17^ *for included studies*

*
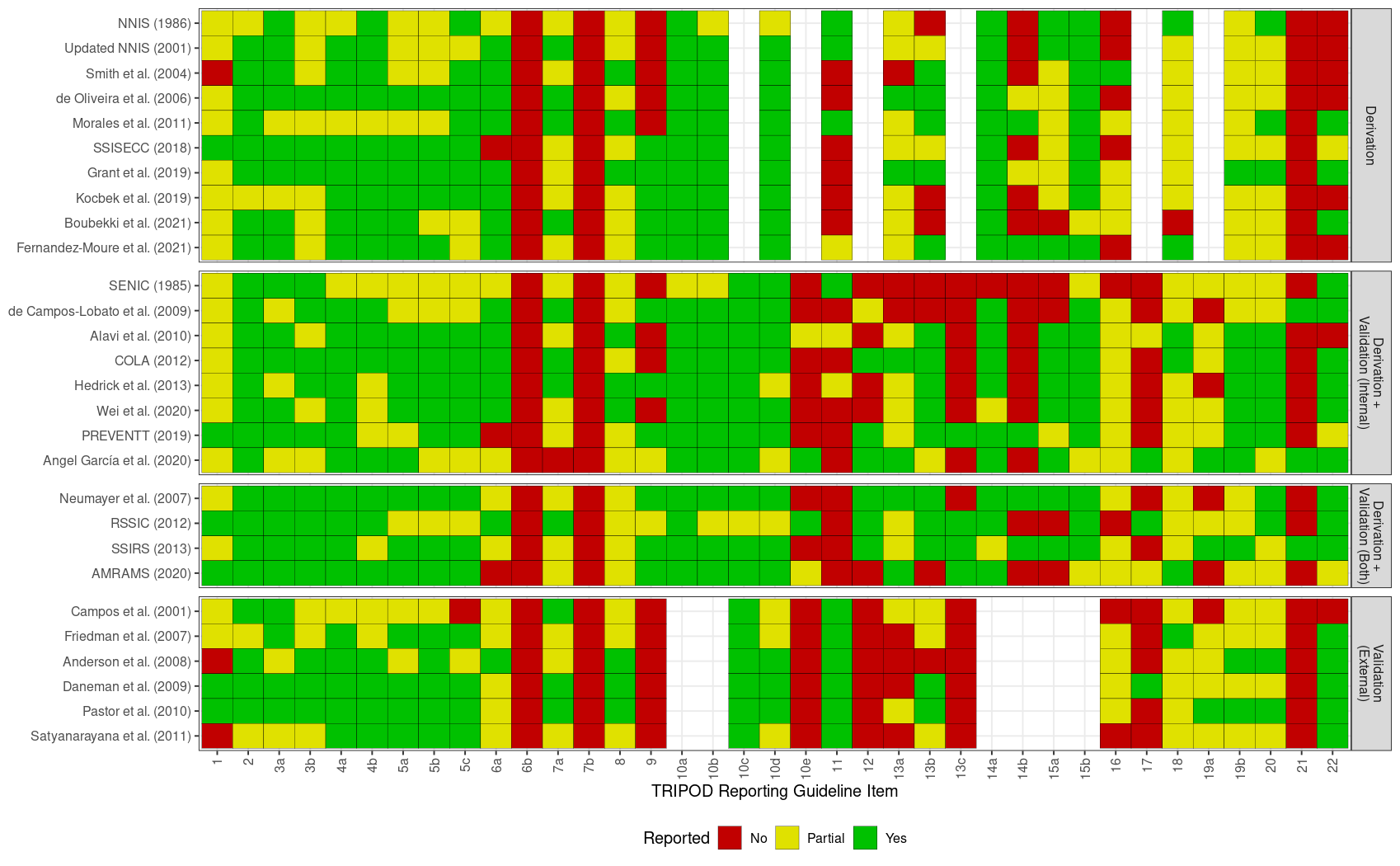
*

## *Supplementary Figure 3: Adherence to PROBAST* ^18^ *quality assessment for included studies*

*
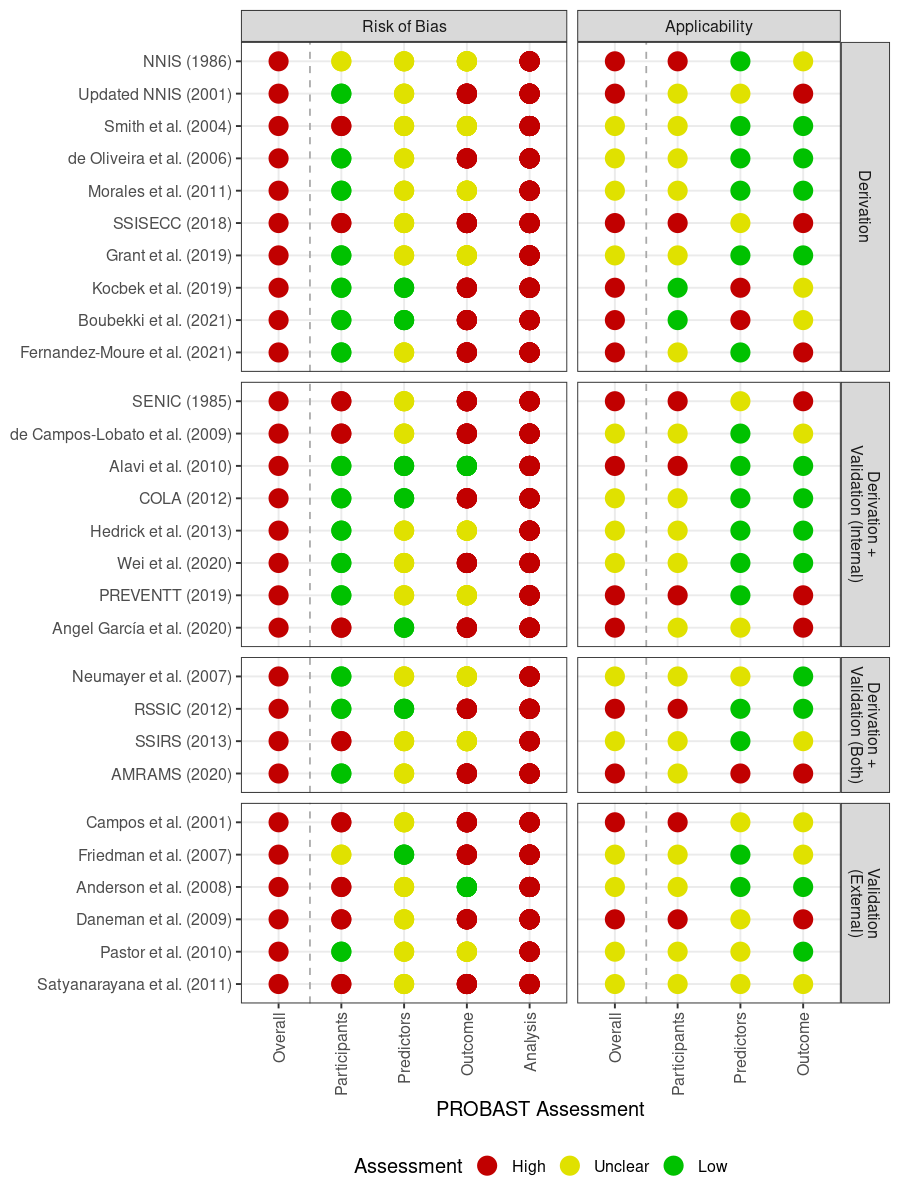
*

## *Supplementary Table 1: Full specification of additional variables included in all original models identified on systematic review.*

| **Score** | **Other Sociodemographic** | **Other Comorbidities** | **Other Preoperative** | **Other Operative** |
| --- | --- | --- | --- | --- |
| SENIC (1985) ^30^ | - | Number | - | - |
| Smith et al. (2004) ^24^ | - | - | - | Hypotension |
| de Oliveira et al. (2006) ^25^ | - | - | - | Procedure risk of SSI |
| Neumayer et al. (2007) ^31^ | - | Alcohol intake, Corticosteroid use, Dyspnoea | Radiotherapy, Total bilirubin | Complexity |
| de Campos-Lobato et al. (2009) ^32^ | - | - | Steriod use, Prior procedure, Radiotherapy, Creatinine | Transfusion |
| Alavi et al. (2010) ^33^ | - | Weight loss | - | - |
| Morales et al. (2011) ^26^ | - | - | - | Destination |
| RSSIC (2012) ^34^ | - | Chemotherapy | - | - |
| Hedrick et al. (2013) ^37^ | - | Alcohol intake, Functional status | Preoperative hematocrit | Intraoperative transfusion, Stoma closure |
| SSIRS (2013) ^36^ | - | Peripheral Vascular Disease (PVD), Metastatic cancer, Corticosteroid use | Preoperative SIRS/Sepsis | General Anaesthesia, Concurrent procedure, Current Procedural Terminology (CPT-3) Score |
| SSISECC (2018) ^27^ | - | - | Obstruction, Electrolyte imbalance, C-reactive protein (CRP) | Transfusion, Thickness of subcutaneous fat, Hypothermia |
| Kocbek et al. (2019) ^43^ | - | - | Alanine aminotransferase (ALAT), Alkaline phosphatase (ALP), Amylase, Aspartate aminotransferase (AST), Total bilirubin, Creatinine, C-reactive protein (CRP), Glucose, Haemoglobin, White cell count (WCC), Potassium, Sodium, Thrombocyte count | - |
| PREVENTT (2019) ^38^ | - | - | - | Indication |
| AMRAMS (2020) ^41^ | Marital status | Weight | Time to operation, Aspartate Aminotransferase (AST), Monocyte count, Total Bilirubin, Potassium, Thrombin time (TT), Direct bilirubin (DBil), INR (international normalized ratio), Sodium, Lymphocyte count, Calcium, Total protein, Glucose, Mean corpuscular haemaglobin (MCH), Alumbin, Postprandial Plasma Glucose, Uric acid, Activated Partial Thromboplastin Time (APTT), Mean corpuscular volume (MCV), Magnesium, Hemoglobin (Hgb) | Anaesthesia |
| Angel García et al. (2020) ^40^ | - | Coagulopathies, Hypertension, Heart failure, Renal insufficiency, Solid tumour | - | - |
| Wei et al. (2020) ^39^ | Ethnicity | - | Mechanism of injury, Base excess, Temperature | Intention, Procedure, Transfusion |
| Boubekki et al. (2021) ^42^ | - | - | Alanine aminotransferase (ALAT), Alkaline phosphatase (ALP), Amylase, Aspartate aminotransferase (AST), Total bilirubin, Creatinine, C-reactive protein (CRP), Glucose, Haemoglobin, White cell count (WCC), Potassium, Sodium, Thrombocyte count | - |
| Fernandez-Moure et al. (2021) ^29^ | - | Weight loss, Radiotherapy, Prior procedure | - | Indication |

## *Supplementary Table 2: Full specification of model evaluation metrics across derivation and validation cohorts.*

| **Score** | **Model** | **Event Rate** | **Discrimination (AUC)** | **Prognostic Accuracy** | **Calibration** | **Other Measure** |  |
| --- | --- | --- | --- | --- | --- | --- | --- |
| SENIC (1985) ^30^ | Derivation ^30^ | Unreported (n=Unreported/58498) | - | - | - | - |  |
|  | Internal validation ^30^ | Unreported (n=Unreported/59352) | - | - | - | - |  |
|  | External Validation ^26^ | 13.8% (n=85/614) | 0.683 (0.63-0.74) | - | - | - |  |
|  | External Validation ^27^ | 7.1% (n=72/1008) | 0.686 | - | - | - |  |
| NNIS (1986) ^12^ | Derivation ^12^ | 2.8% (n=2376/84691) | - | - | - | G&K = 0.44 (SE = 0.015) |  |
|  | External Validation ^44^ | 6.8% (n=632/9322) | - | - | - | G&K = 0.49 |  |
|  | External Validation ^25^ | 24.5% (n=149/608) | 0.627 (0.575-0.678) | - | H&L = 0.652 | - |  |
|  | External Validation ^45^ | Unreported (n=Unreported/17899) | - | - | - | G&K = Positive |  |
|  | External Validation ^31^ | 4.3% (n=7027/163378) | 0.6166 | - | - | - |  |
|  | External Validation ^46^ | 5.5% (n=284/5144) | - | - | - | G&K = 0.38 (95% CI: 0.24-0.53) |  |
|  | External Validation ^47^ | 8.1% (n=38131/469349) | 0.59 | - | - | - |  |
|  | External Validation ^26^ | 13.8% (n=85/614) | 0.707 (0.65-0.76) | - | - | - |  |
|  | External Validation ^48^ | 13.7% (n=137/1000) | - | - | - | - |  |
|  | External Validation ^34^ | 24.3% (n=122/503) | 0.63 | - | - | - |  |
|  | External Validation ^36^ | 3.9% (n=14227/363040) | 0.641 | - | - | - |  |
|  | External Validation ^27^ | 7.1% (n=72/1008) | 0.732 | - | - | - |  |
|  | External Validation ^41^ | 1.1% (n=45*/4014) | 0.651 | SEN = 0.372, SPE = 0.930 | - | - |  |
| Updated NNIS (2001) ^13^ | Derivation ^13^ | 1.0% (n=449/42815) | - | - | - | - |  |
|  | External Validation ^49^ | 19.3% (n=95/491) | 0.59 | - | H&L = 0.81 | - |  |
| Smith et al. (2004) ^24^ | Derivation ^24^ | 25.6% (n=45/176) | 0.7 | - | H&L = 0.719 | - |  |
| de Oliveira et al. (2006) ^25^ | Derivation ^25^ | 24.5% (n=149/608) | 0.732 (0.685-0.779) | - | H&L = 0.995 | - |  |
| Neumayer et al. (2007) ^31^ | Derivation ^31^ | 4.4% (n=3555/81638) | 0.7165 | - | - | - |  |
|  | Internal validation ^31^ | 4.2% (n=3464/81666) | 0.7001 | - | - | - |  |
| de Campos-Lobato et al. (2009) ^32^ | Derivation ^32^ | 3.1% (n=385/12373) | - | - | - | - |  |
|  | Internal validation ^32^ | 3.6% (n=343/9521) | - | - | - | - |  |
| Alavi et al. (2010) ^33^ | Derivation ^33^ | 22.6% (n=899/3978) | 0.644 | - | - | - |  |
|  | Internal validation ^33^ | 24.7% (n=783/3171) | 0.624 | - | - | - |  |
| Morales et al. (2011) ^26^ | Derivation ^26^ | 13.8% (n=85/614) | 0.798 (0.75-0.85) | - | - | - |  |
| COLA (2012) ^35^ | Derivation ^35^ | 21.3% (n=114/534) | 0·729 (0·675 to 0·783) | - | - | - |  |
|  | Internal validation ^35^ | 21.3% (n=114/534) | 0·703 (0·644 to 0·761) | - | - | - |  |
|  | External Validation ^28^ | 12.9% (n=3789/29324) | 0.64 (0.63-0.65) | - | - | - |  |
|  | External Validation ^28^ | 5.8% (n=130/2237) | 0.62 (0.58-0.67) | - | - | - |  |
|  | External Validation ^28^ | 9.3% (n=1345/14516) | 0.60 (0.58-0.61) | - | - | - |  |
| RSSIC (2012) ^34^ | Derivation ^34^ | 24.3% (n=122/503) | - | - | - | - |  |
|  | Internal validation ^34^ | 24.3% (n=122/503) | 0.70 (0.65-0.75) | - | - | - |  |
| Hedrick et al. (2013) ^37^ | Derivation ^37^ | 9.3% (n=1719/18403) | - | - | - | - |  |
|  | Internal validation ^37^ | 9.3% (n=1719/18403) | 0.64 | - | “well calibrated across most of the range of probabilities of infection, with poor calibration only in the range of extremely high risk, for which there are very few patients” | - |  |
| SSIRS (2013) ^36^ | Derivation ^36^ | 3.9% (n=7128*/181894) | 0.772 | - | - | - |  |
|  | Internal validation ^36^ | 3.9% (n=7099*/181146) | 0.800 (0.795-0.805) | - | - | - |  |
| SSISECC (2018) ^27^ | Derivation ^27^ | 7.1% (n=72/1008) | 0.82 | - | - | - |  |
| Grant et al. (2019) ^28^ | Derivation ^28^ | Unreported (n=Unreported/46320) | 0.65 (0.64-0.66) | - | - | - |  |
| Kocbek et al. (2019) ^43^ | Derivation ^43^ | 20.1% (n=183/909) | 0.954 (0.951-0.957) | SEN = 0.836 (0.824-0.847), SPE = 0.919 (0.915-0.924),  PPV = 0.724 (0.710-0.739), NPV = 0.957 (0.954-0.960) | - | - |  |
| PREVENTT (2019) ^38^ | Derivation ^38^ | 21.1% (n=366/1737) | - | - | - | - |  |
|  | Internal validation ^38^ | 21.1% (n=366/1737) | 0.69 | - | Intercept = -0.067, Slope = 0.947 | - |  |
| Wei et al. (2020) ^39^ | Derivation ^39^ | 14.6% (n=120*/820*) | 0.854 (0.81-0.89) | - | - | - |  |
|  | Internal validation ^39^ | 14.8% (n=52/351) | 0.78 (0.71-0.85) | - | - | - |  |
| AMRAMS (2020) ^41^ | Derivation - LASSO ^41^ | 1.1% (n=199*/17597) | 0.856 (0.852-0.860) | - | - | - |  |
|  | Internal validation - LASSO ^41^ | 1.1% (n=45*/4014) | 0.816 | SEN = 0.674, SPE = 0.842 | - | - |  |
|  | Derivation - CNN ^41^ | 1.1% (n=199*/17597) | 0.889 (0.886-0.892) | - | - | - |  |
|  | Internal validation - CNN ^41^ | 1.1% (n=45*/4014) | 0.878 | SEN = 0.837, SPE = 0.869 | - | - |  |
| Angel García et al. (2020) ^40^ | Derivation ^40^ | 7.3% (n=463/6325) | 0.62 (0.60-0.65) | - | H&L = 0.57 | - |  |
|  | Internal validation ^40^ | 7.3% (n=463/6325) | 0.62 (0.60-0.65) | - | H&L = non-signficant | - |  |
| Boubekki et al. (2021) ^42^ | Derivation ^42^ | 20.1% (n=183/909) | 0.991 (0.984-0.998) | SEN = 0.874 (0.848-0.900), SPE = 0.987 (0.974-1.000),  PPV = 0.979 (0.963-0.995), NPV = 0.934 (0.921-0.947) | - | - |  |
| Fernandez-Moure et al. (2021) ^29^ | Derivation ^29^ | 13.3% (n=632/4738) | 0.802 | - | - | - |  |
| Values within brackets represent 95% confidence intervals. **Abbreviations**: H&L = Hosmer–Lemeshow test, G&K = Goodman–Kruskal γ correlation coefficient, SEN = Sensitivity, SPE = Specificity, PPV = Positive Predictive Value, NPV = Negative Predictive Value. | | | | | | | |
